# Annotations

**Published:** 1897-06-26

**Authors:** 


### Annotations.

### The Recent Plague in Bombay.

The death-rate in Bombay has at last returned to the normal; in fact it has now become lower than the mean of the corresponding weeks in the preceding five years, which is probably due to the population having been to a considerable extent reduced. In any case the great outbreak of plague which has held the city in its clutches for the last eight months may now be looked upon as practically at an end, and all we can do is to reckon up the losses which have been incurred. From the commencement of the epidemic the *Times of India* has maintained that the official lists showed but a small proportion of the actual mortality. In this we think it has been right. Considering the tendency to concealment of the true cause of death which admittedly has prevailed from the very first, and in view of the fact—the grim fact which could not be hidden—that the general death-rate during the epidemic increased far beyond what was accounted for by the official returns of the deaths from plague, we cannot but feel that the only plan by which the fatality of the disease could be even approximately estimated was by subtracting the average mortality of the weeks in question during the last few years from the actual total mortality recorded, and taking the difference as showing the deaths from plague. If this is done, it shows that between September 29th and May 25th nearly 21,000 persons died of plague in Bombay, notwithstanding the enormous emigration which had taken place before the height of the epidemic. This is a serious loss, amounting to nearly one in every thirty-eight of the population. Still, it might have been worse. Great as this mortality seems, it is far away less than that which obtained in many of the epidemics of the middle ages, and very much less than that in Canton a few years ago.

### Public Gardens.

The difficulties which have arisen in the acquisition of the Postmen's Park, a small but just at present a delightfully green oasis amid the tall buildings in the City, serve to remind us of the great importance from a public health point of view of the little gardens and open spaces scattered among the central districts in London which are maintained by the Metropolitan Public Gardens Association. We hear a great deal about the necessity of securing open spaces around London, so that the growing outer ring may not become an even more monotonous expanse of streets and buildings than it is at present, and we can quite sympathise with those who would surround the existing town, while there is yet time, with a circle of noble parks. We cannot, however, but see that desirable as it is to interpose large air spaces between the different districts, it is a matter of far greater moment that small gardens and playgrounds should be interspersed among the streets themselves. A little fresh air near at hand is really more valuable to town dwellers than a great deal further off. We have only to look at our great parks, and especially at such large suburban open spaces as Hampstead Heath, to see how little they are used as breathing grounds by the working classes, except on high days and holiday. They are too far off, they are places to go to for the day much as Brighton is, but they do not enter into the daily lives of the mass of town dwellers. It is otherwise, however, with the little gardens, the playgrounds, the converted graveyards, and other small spaces which, under the fostering care of the Metropolitan Public Gardens Association, are coming into being in so many of the more densely populated districts. These are a constant influence for good in the districts where they have been established. A good deal has been said lately about the desirability of enlarging Hampstead Heath and of keeping certain properties out of the hands of the builder. Well, on æsthetic grounds this may be perfectly right, and we have no desire to quarrel with those who wish to spend their money in so good a cause. But when it becomes a question of appealing to others, and basing the request for funds upon the plea that it is for the good of the public health that such an addition should be made, we feel bound to say that, so far as public health is concerned, the money would be far more usefully employed in the establishment of new centres of fresh air, and especially in the development of small gardens and playgrounds in populous places, than in the enlargement of a heath which is already ample for all sanitary purposes, and, so far as the masses are concerned, is only used by them a few times a year, and then chiefly as a fair-ground.

### The Responsibilities of Poor Law Guardians

A very curious application was made at the Thames Police-court the other day. According to the report in the *Times*, a labouring man told the magistrate that being in want of shelter he went to the casual ward of St. George's-in-the-East. The next morning he was put on stone-breaking, and, in consequence, had lost the sight of his right eye. Could he claim compensation? The magistrate inquired whether he did not have a guard, and the warrant officer stated that at most of the casual wards the men who were put on stone-breaking were supplied with goggles. He did not know about St. George's. The applicant said, however, he had no goggles, and handed in a certificate from the eye hospital at Moorfields. The magistrate, Mr. Mead, is reported to have said that it was very probable the Guardians were liable if they did not provide proper protection. Once inside, the applicant had no option but to do the work set him. He had better go to the Local Government Board and ask if there was any regulation by that body for the provision of guards. If after that he returned to the Court he would see what could be done. This opens up a somewhat serious prospect for the Guardians. There are many things besides breaking stones in regard to which a man has no option when once he enters a workhouse; and if it is to be admitted that the Guardians are to be liable for any evils which result from want of care in the arrangements for their performance, we can easily imagine that many very curious claims for compensation may crop up. The question how far ophthalmia is preventable by the exercise of proper care may become a very serious one if damages are to be claimed for damaged eyes.

